# Applying Complex Network and Cell-Cell Communication Network Diagram Methods to Explore the Key Cytokines and Immune Cells in Local Acupoint Involved in Acupuncture Treating Inflammatory Pain

**DOI:** 10.1155/2020/2585960

**Published:** 2020-07-29

**Authors:** Kuo Zhang, Xue Zhao, Shasha Ding, Yangyang Liu, Yuan Xu, Yawen Yan, Shouhai Hong, Fuming Yang, Shenjun Wang, Zhifang Xu, Yongming Guo, Yi Guo, Guangchang Pang, Jiang Wang, Xinmeng Guo, Miao Zhao

**Affiliations:** ^1^Academy of Medical Engineering and Translational Medicine, Tianjin University, Tianjin 300072, China; ^2^Research Center of Experimental Acupuncture Science, Tianjin University of Traditional Chinese Medicine, Tianjin 301617, China; ^3^Department of Acupuncture and Physical Therapy, Tianjin Nankai Hospital, Tianjin 300100, China; ^4^Acupuncture Department, Zhejiang Provincial Hospital of TCM, Hangzhou, Zhejiang 310006, China; ^5^Jiangsu Key Laboratory of Molecular Medicine, Medical School of Nanjing University, Nanjing, Jiangsu 210093, China; ^6^School of Traditional Chinese Medicine, Tianjin University of Traditional Chinese Medicine, Tianjin 301617, China; ^7^Tianjin Key Laboratory of Food Biotechnology, Faculty of Biotechnology and Food Science, Tianjin University of Commerce, Tianjin 300134, China; ^8^School of Electrical Engineering and Automation, Tianjin University, Tianjin 300072, China

## Abstract

Manual acupuncture (MA) can effectively treat a variety of diseases, but its specific mechanism remains unclear. The “acupoint network” activated by MA participates in MA signal transduction, in which immune-related cells and cytokines play an important role. However, which cells and cytokines in the acupoint have changed after MA? What is the network relationship between them? Which cells and cytokines may play the most important role in MA effect? These problems are unclear. In this study, on the basis of affirming the analgesic, detumescence, and anti-inflammatory effect of MA, the concentration of 24 cytokines in ST36 acupoint in rats with inflammatory pain after MA treatment was detected by multiplex immunoassay technology. Then, using statistical and complex network and cell-cell communication (CCC) network diagram method to analyze the detected data depicts the network relationship between the cytokines and related cells objectively and establishes cytokine connection network and CCC network, respectively. The results showed that MA reinforced communication intensity between cells while reducing the overall correlation intensity. On this basis, the key cytokines and key cells at three MA time-points were screened out, cytokines IL-6, MCP-1, fibroblasts cell, and monocyte macrophage screened by the three methods at three MA time-points might be the key cytokines or key cells. After that, we detected the macrophages in ST36 acupoint by flow cytometry and immunofluorescence and found that the relative amount of macrophages increased significantly after MA, especially the macrophage of the dermis of skin. This study provided a basis for revealing the initiated mechanism of MA effect.

## 1. Introduction

Manual acupuncture (MA) has been practiced in China for more than 3000 years and has gained increasing popularity and acceptance in clinical practice worldwide [1, 2]. However, its mechanism has not yet been fully elucidated [3].

Increasing evidence shows the acupoint is the initial and key regulatory site of the MA effect [4, 5]. The needle manipulation process, through mechanical stimulation (microtrauma), can cause afferent nerve excitation [6], connective tissue deformation [7], ion channel on or off [8] in the acupoint, which promotes the release of multiple neurotransmitter, endocrine hormones, and immune factors [9, 10]. These substances interacted with each other, constituting the local “acupoint network” [11], which initiate and transmit the MA information.

Our previous studies found that some immune-related cells and cytokines in acupoint changed significantly after MA. The concentrations of TLR4, E-selectin, L-selectin, and cytokines IL-1*β*, IL-6, IL-8, and TNF-*α* in the acupoint were significantly increased within a certain time after MA [12, 13], and the number of mast cells and their degranulation rate in the acupoint increased significantly and participated in the MA effect. We also observed that the local tissue was damaged and degenerated and necrotic and neutrophil infiltration in the acupoint by twisting needles [12]. It is well known that immune cells communicate with each other by cytokines, forming an immune network [14]. So, which cells and cytokines in the acupoint have changed after MA? What is the network relationship between these cells and cytokines? Which cells and cytokines may play the most important role in MA effect? Are these key cells and cytokines the same at different MA time-points? These problems are unclear and need to be further studied.

In our previous study, we have demonstrated that MA has an analgesic, detumescence, and anti-inflammatory effect on CFA-induced inflammatory pain rats and tried to definite the key cells and key cytokines in rat serum responded to MA by complex network and cell-cell communication (CCC) network method. In this study, we focused on the immune function changes of the local acupoint after MA. Firstly, we detected the concentrations of 24 cytokines of ST36 acupoint in CFA-induced inflammatory pain rats after MA treatment by multiplex immunoassay technology and analyzed the data using statistical and complex network and cell-cell communication network diagram method, in order to depict the network relationship between the cytokines and related cells objectively and establish cytokine connection network and CCC network, respectively. On this basis, the key cytokines and key cells of the acupoint at three time-points were selected, and the key cells were detected by flow cytometry and immunofluorescence for localization and quantitative detection. This study hopes to provide an accurate and reliable scientific basis for revealing the acupoint initiation mechanism of MA effect.

## 2. Materials and Methods

### 2.1. Animals

In this experiment, adult male Wistar rats (weight: 180 ± 20 g, 6 to 8 weeks old) were obtained from the Institute of Hygiene and Environmental Medicine, Academy of Military Medical Sciences, PLA. Rats were housed on a 12 h light/dark cycle and were provided food and water ad libitum for 1 week prior to the animal experiments. All animal procedures were approved by the Animal Ethics Committee of Tianjin University of Traditional Chinese Medicine in China (TCM-LAEC2012010) and carried out in accordance with the International Guide for the Care and Use of Laboratory Animals.

### 2.2. Experimental Procedures

**Figure 1:**
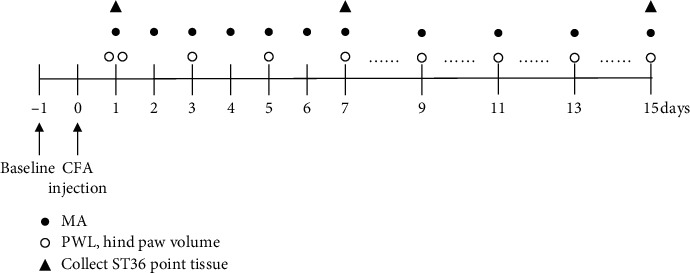
Outline of the experimental protocol.

The outline of the study is given in Figure 1. All rats were randomly divided into 3 groups: control group, CFA group, and CFA + MA group (*n* = 20 per group). Rats were received intraplantar injections (i.pl.) of complete Freund's adjuvant (CFA, Sigma, San Diego, CA, USA) or normal saline on day 0. The ipsilateral nociceptive thresholds (paw withdrawal latency, PWL) and swelling of the paw were measured on day 1, day 1 (before and after CFA/normal saline injection) and days 1–15 (every other day and 0.5 h after MA). MA treatment was given once a day for 7 consecutive days (days 1–7) and then given every other day (days 8–15), for a total of 11 sessions. To detect cytokines levels and conduct a localization and quantitative analysis of macrophages, the acupoint tissues were collected after behavioral tests on three different MA time-points: day 1, day 7, day 15 (evaluate the cytokines *n* = 6-7, macrophage location detection *n* = 3, and quantitative detection *n* = 3). All experimental tests were performed in a double-blind manner.

### 2.3. Induction of CFA Model

Intraplantar inflammation in the right hind paw was induced by i.pl. administration of 100 *μ*L CFA in CFA group and CFA + MA group. As a control, 100 *μ*L of normal saline was injected in the control group [15].

### 2.4. Manual Acupuncture Treatment

Rats were subjected to fixation using the soft cloth fixation method. Acupuncture needles (0.35 mm in diameter and 25 mm in length, Hanyi TCM, Beijing, China) were inserted to a depth of 5–7 mm at bilateral ST36 (Zusanli) acupoints. The needles were rotated at a rate of 3 spins per second bidirectionally, one spin consisted of clockwise rotation of 180° and a counterclockwise rotation of 180° for 2 min after Deqi, mild reinforcing, and attenuating [16]. The needles were manipulated every 5 min for a 30 min session. Rats in control and CFA groups were only subjected to fixation using the soft cloth without other interventions.

### 2.5. Measurement of Thermal Hyperalgesia

Thermal hyperalgesia was assessed by determination of PWL in response to a thermal stimulus (radiant heat) administered via a plantar test analgesia meter BME-410 C heat pain stimulation instrument (Chinese Academy of Medical Sciences Institute of Biomedical Engineering, Tianjin, China). Rats were placed into test enclosures, with a glass plate underneath, to acclimatize for 30 min. The radiant heat source was then applied to the plantar surface of the right hind paw from underneath the glass plate. A light beam intensity that elicited baseline paw withdrawal latencies of 15–23 s was used. A maximum limit of 30 s was imposed in order to prevent tissue damage in the absence of a withdrawal response. The PWL was established by averaging the latency of 3 tests with a 5 min interval between each test [17].

### 2.6. Measurement of Swelling of Paw

The swelling of rat's right hind paw was measured by volumetric method [18] with a self-made foot volume meter [19]. The hind paw was immersed in a chamber containing phosphate buffered saline (PBS) up to the boundary between hairy and nonhairy skin. The volume displacement represented the hind paw swelling and was determined by two observers as previously described [16].

### 2.7. Local Acupoint Tissue Sample Collection

One day before sample collection, the hair on the legs was removed from the skin using electronic hair clipper. After PWL measurement at day 1, day 7, and day 15, rats were anesthetized with a peritoneal injection of pentobarbitone sodium (50 mg/kg), then the rats were killed by cervical dislocation, and the tissues located in the right ST36 (1 cm in diameter and 0.5 cm thick, consisting of skin and subcutaneous and muscle tissues) were collected immediately with a scalpel.

### 2.8. Cytokines Detection

6-7 rats in each group were randomly selected for 24 cytokines detection of the ST36 acupoint tissue. All tissues were triturated into homogenate with liquid nitrogen, followed by centrifugation at 2000 r/min for 10 min at 4°C to get supernatants. 23 of the 24 cytokines were detected by the Bio-Plex ProTM Rat Cytokine 24-Plex Assay kit (Bio-Rad Laboratories, Hercules, CA, USA). 23 cytokines are as follows: IL-1*α*, IL-1*β*, IL-6, IL-7, IL-18, TNF-*α*, IL-2, IL-12, IFN-*γ*, IL-4, IL-5, IL-10, IL-13, IL-17, CXCL 1, MCP-1, RANTES, MIP-1*α*, MIP-3, GM-CSF, G-CSF, M-CSF, and VEGF. Cytokine CRP protein was detected by ELISA method using a commercial kit (Beijing Sino-uk Institute of Biological Technology, Beijing, China). All the detections were performed according to the manufacturer's instructions.

### 2.9. Immunofluorescence Staining

Three rats in each group were randomly selected for localization detection of macrophages in the ST36 acupoint tissue. All tissues were fixed in 4% paraformaldehyde for 24 h; then the tissues were soaked in 20% sucrose for at least 48 h after being washed with distilled water. The OCT-embedded blocks were sectioned at 4 *μ*m thickness. Sections from each group were rinsed in PBST (0.05% Tween 20 in PBS) and blocked for 1h with blocking solution (10% horse serum in PBST) at room temperature. The blocking solution was removed, and the primary antibody Anti-CD68 antibody (1 : 200, Abcam, San Francisco, USA) was diluted in 10% horse serum and added to the sections. These solutions were incubated overnight at 4°C. The antibody solution was removed and the sections were washed with PBST. The sections were incubated with Alexa Fluor 488 Goat Anti-Rabbit IgG (diluted 1 : 400, Abcam, San Francisco, USA) for 2 h in a dark room and mounted using mounting medium with 4′,6-diamidino-2-phenylindole (DAPI) for 8 min. The CD68-positive signals were detected using fluorescence microscope (Olympus BX51).

### 2.10. Flow Cytometry

Three rats in each group were randomly selected for quantitative detection of macrophages in the ST36 acupoint tissue. Tissue samples were cut into 1 to 2 mm pieces. Fragments were digested for 1 h at 37°C with 9.75 ml RPMI medium1640/each tissue sample containing 10 mg collagenase, 1 mg hyaluronate, and 0.25 ml HEPES (Sigma-Aldrich, St. Louis, MO, USA). The digested fragments were pressed through a 70 *μ*m nylon cell strainer (BD Falcon, Franklin Lakes, NJ, USA) to remove particles. Macrophage was stained with PE Mouse Anti-Rat CD45, APC Mouse Anti-Rat CD11b (BD Biosciences, San Jose, CA, USA), and FITC Mouse Anti-Rat CD68 (AbD Serotec, Kidlington, UK). Cells stained with isotype-matched irrelevant control antibodies and unstained cells were used as negative controls. Cells were detected by a Attune™ NxT Acoustic Focusing Cytometer (Thermo Fisher Scientific, San Jose, CA, USA) and analyzed by Attune NxT software (Thermo Fisher Scientific).

### 2.11. Data Analysis

The data of PWL, swelling of paw, macrophages obtained from flow cytometry, and immunofluorescence were analyzed by statistical analysis method. The data about cytokines concentrations was analyzed by 3 methods, including statistical analysis, complex network analysis, and CCC network diagram method. 24 cytokines were classified and statistically analyzed according to chemokine, cytokine of innate immunity, adaptive immunity, and several growth factors, and then based on the detection data, the immune cytokine connection networks and directed weighted CCC networks were established by complex network and CCC network diagram methods, for getting the key cells and key cytokines of the acupoint in MA treatment.

### 2.12. Statistical Analysis

Statistical analyses were performed using SPSS 19.0 software (SPSS Inc., Chicago, IL, USA). All statistical data were presented as the mean ± SEM. *P* values <0.05 were considered statistically significant.

PWL and swelling of paw were analyzed by repeated measures analysis of variance, and multivariate analysis of variance was used for intergroup statistical analysis. Cytokines levels of the ST36 acupoint tissues and the data of macrophages were analyzed by one-way ANOVA. LSD method was used if accorded with homogeneity test of variance, while Dunnett's T3 method was used if not accorded with homogeneity test of variance. If data were not normally distributed, they were transformed using commonly accepted methods or analyzed with a nonparametric test. The cytokines with statistical significance between groups (CFA + MA group compared with CFA group) were identified as the possible key cytokines in MA regulating acupoint network. All figures were generated using GraphPad Prism (GraphPad Software, La Jolla, CA).

### 2.13. Complex Network Analysis

For more direct and visual analysis of the changes of cytokines levels of ST36 acupoint tissues induced by MA and screening the key cytokines, the data of 24 cytokines in the acupoint after MA at three time points (day 1, day 7, and day 15) were analyzed by complex network methods, which consist of 3 steps and have been described in previous study [18]. In Step 1, based on the detection data of 24 cytokines, correlation coefficients between cytokines were calculated by Pearson correlation formula which was constructed by MATLAB software (Natick, America), the cytokines with correlation coefficients ∈ [−1, −0.8], [0.8, 1] were filtered to construct the cytokines correlation network. In Step 2, the sum of cytokines correlation strength in different groups (Connection network_str_) was obtained by adding the absolute value of the screened cytokines correlation coefficient, which can be used to depict the overall correlation intensity of cytokines at ST36 acupoint in rats under different status. In Step 3, the screened cytokines were sorted by 3 kinds of complex network methods (i.e., node degree, node strength correlations, and node clustering coefficient. ① Node degree: the number of edges connected to each node, a node of larger degree meaning it was more important in the network. ② Node strength correlations: the sum of absolute values of correlation coefficients of each node, a node of higher strength correlations meaning it was more important in the network. ③ Node clustering coefficient: it represented the possibility of connections between the other nodes that were connected to this one node, a node of lower strength correlations meaning the other nodes are with low possibility of connections except this one node, this also suggested that the node was important in the network.) that could measure the importance of nodes in the network. Two or more methods in the first three nodes obtained by analysis with 2 or 3 methods mentioned above were considered as the key cytokines screened by the complex network analysis.

### 2.14. Cell-Cell Communication (CCC) Network Diagram Method

We screened the key cells of “acupoint network” after MA using the CCC network diagram method. The communication effect between all the cells was calculated according to the following formula: Ecc=∑_*i*=1_^*n*=24^*S*_*i*_*F*_*i*_*p*′, the meaning of each letter or word of the formula was described in previous study [16]. The line between the cells represented the communication between the cells. The thickness of lines, quantified according to Ecc values, represented the total stimulating intensity of cytokines on the target cells. Secretory cell_n_ represented the secretory density (secretory cell_*n*_ = En1 + En2 + En3 + En4 + · · · + Enn) of the cell. For instance, secretory cell_1_ = E11 + E12 + E13 + E14 + · · · + E1n). Target cell_*n*_ represented the target density (target cell_*n*_ = E1n + E2n + E3n + E4n + · · · + Enn). For instance, Target cell_1_ = E11 + E21 + E31 + E41 + · · · + En1). Communication network_sd_ represented the whole immune network density between cells (communication network_sd_ = (E11 + E12 + E13 + E14 + · · · + E1n) + · · ·(En1 + En2 + En3 + En4 + · · · + Enn)). Cell_sd_ represented the total of absolute density including secretory and target densities (Cell_sd_ = Abs (secretory cell_n_) + Abs (target cell_n_). The cells with the maximum value of secretory cell_*n*_, target cell_*n*_, and cell_sd_ were considered as the key cells screened by the CCC network diagram method.

## 3. Results

### 3.1. The Analgesic and Anti-Inflammatory Effect of MA on Rats with CFA-Induced Inflammation

**Figure 2:**
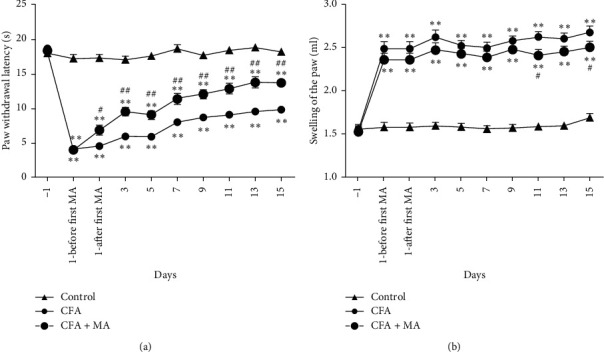
The antinociceptive and anti-inflammatory effect of MA on CFA-induced inflamed paw of the rats. (a) Effect of MA on thermal hyperalgesia at different time-points (*n* = 20 rats in each group). (b) Effect of MA on hind paw swelling at different time-points (*n* = 20 rats in each group). All data are shown as the mean ± SEM, ^*∗*^*P* < 0.05 and ^*∗∗*^*P* < 0.01, versus control group; ^#^*P* < 0.05 and ^##^*P* < 0.01, versus CFA group.

**Figure 3:**
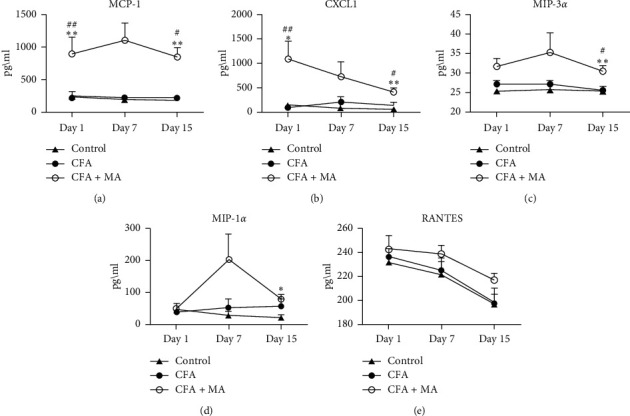
MA regulated the levels of chemokines in the ST36 acupoint. The levels of MCP-1 (a), CXCL-1 (b), MIP-3*α* (c), MIP-31 (d), and RANTES (e) in ST36 acupoint of the rats receiving injection of saline/CFA and treated with or without MA in day 1, day 7, and day 15 (*n* = 5–7). Data are expressed as the mean ± SEM, ^*∗∗*^*P* < 0.01 and ^*∗*^*P* < 0.05 versus control group. ^##^*P* < 0.01 and ^#^*P* < 0.05 versus CFA group.

**Figure 4:**
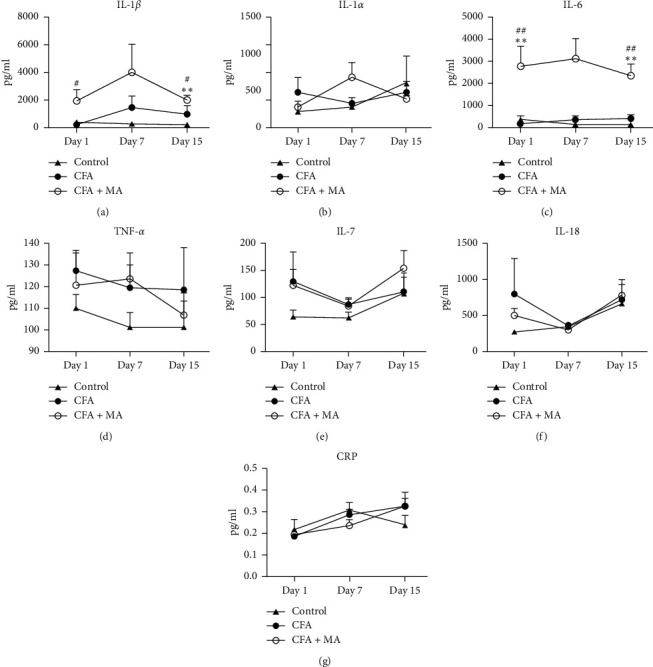
MA regulated the levels of innate immune cytokines in the ST36 acupoint. Levels of IL-1*β* (a), IL-*α* (b), IL-6 (c), TNF-*α* (d), IL-7 (e), L-18 (f), and CRP (g) in ST36 acupoint of the rats receiving injection of saline/CFA and treated with or without MA in day 1, day 7, and day 15 (*n* = 5–7). Data are expressed as the mean ± SEM, ^*∗∗*^*P* < 0.01 versus control group, ^#^*P* < 0.05 and ^##^*P* < 0.01 versus CFA group.

**Figure 5:**
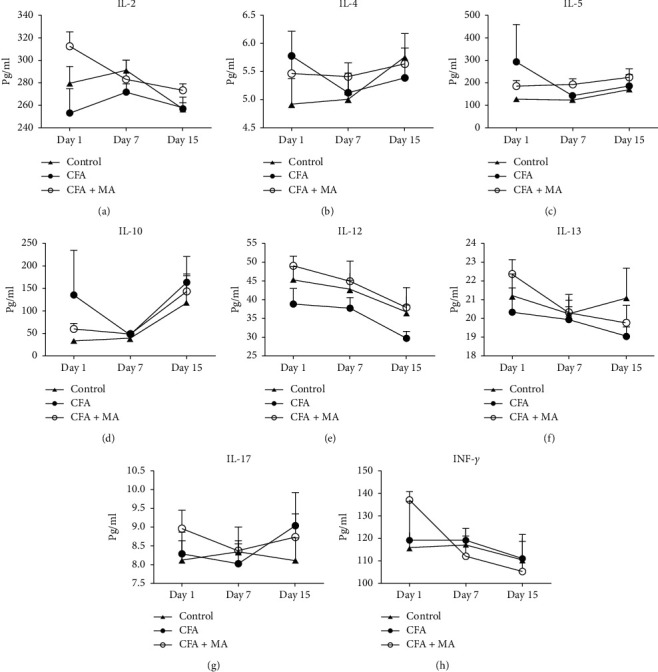
MA did not regulate the levels of adaptive immune cytokines in the ST36 acupoint. Levels of IL-2 (a), IL-4 (b), IL-5 (c), IL-10(d), IL-12 (e), IL-13(f), IL-17 (g), and IFN-*γ* (h) in ST36 acupoint of the rats receiving injection of saline/CFA and treated with or without MA in day 1, day 7, and day 15 (*n* = 5–7). Data are expressed as the mean ± SEM.

**Figure 6:**
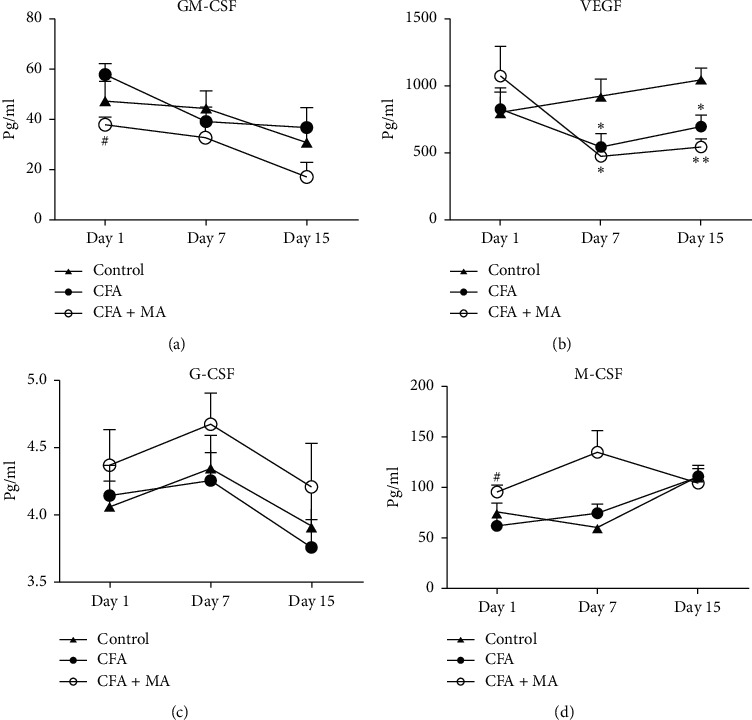
MA regulated the levels of several growth factors in ST36 acupoint. Levels of GM-CSF (a), VEGF (b), G-CSF (c), and M-CSF (d) in ST36 acupoint of the rats receiving injection of saline/CFA and treated with or without MA in day 1, day 7, and day 15 (*n* = 5–7). Data are expressed as the mean ± SEM, ^#^*P* < 0.05 versus CFA group.

The outline of the study is given in Figure 1. In the CFA group, PWLs of the inflamed paw to radiant heat were significantly reduced from post-CFA injection (day 1) to day 15 compared to that of the control group (*n* = 20, ^*∗∗*^*P* < 0.01 vs. control group, Figure 2(a)). A persistent swelling on the paw including ankle joint was observed from day 1 (*n* = 20, ^*∗∗*^*P* < 0.01 vs. control group, Figure 2(b)) through 15 days after CFA injection, and the maximal swelling was shown on day 15. MA at ST36 significantly increased PWL from day 1 (after first MA) to 15 days (^##^*P* < 0.05 vs. CFA group Figure 2(a)) and alleviated the paw swelling at day 11 (^#^*P* < 0.05 vs. CFA group) and day 15 (^#^*P* < 0.05 vs. CFA group, Figure 2(b)).These results indicated that MA had an analgesic effect on inflammatory pain in CFA rats and had the effects of anti-inflammatory and detumescence on CFA-induced hind paw swelling (figure 3‐6).

### 3.2. MA Regulated the 24 Cytokines Expressions in the ST36 Acupoint of the Rats with CFA-Induced Inflammation

  ① Changes of the chemokine expressions in the ST36 acupoint of the rats during MA treatment. To investigate the modulation of MA on chemokine, the levels of MCP-1, CXCL1, MIP-1*α*, MIP-3*α*, and RANTES in the ST36 acupoint tissue were determined by multiplex immunoassay. The results showed that in day 1, day 7, and day 15, the concentrations of chemokines in CFA group did not change significantly compared with the control group. Compared with the control group and CFA group, MA could significantly increase the concentrations of chemokine MCP-1 (day 1, ^*∗∗*^*P* < 0.01 vs. control group, ^##^*P* < 0.01 vs. CFA group; day 15, ^*∗∗*^*P* < 0.01 vs. control group, ^#^*P* < 0.05 vs. CFA group, Figure 3(a)), CXCL1(day 1, ^*∗*^*P* < 0.05 vs. control group, ^##^*P* < 0.01 vs. CFA group; day 15, ^*∗∗*^*P* < 0.01 vs. control group, ^#^*P* < 0.05, Figure 3(b)), and MIP-3*α* (day 15, ^*∗∗*^*P* < 0.01 vs. control group, ^#^*P* < 0.05 vs. CFA group, Figure 3(c)) in the local ST36 acupoint.
  ② Changes of the levels of innate immune cytokines in the ST36 acupoint of the rats during MA treatment. IL-1*β*, IL-*α*, IL-6, TNF-*α*, IL-7, L-18, and CRP are some commonly encountered cytokines of innate immunity.  Figure 4 showed that IL-1*β* and IL-6 in CFA + MA group were elevated significantly on day 1 and day 15 compared to those of both control group and CFA group (day 1, ^#^*P* < 0.05 vs. CFA group; day 15, ^*∗∗*^*P* < 0.01 vs. control group, ^#^*P* < 0.05 vs. CFA group, Figure 4(a)) (day 1 and day 15, ^*∗∗*^*P* < 0.01 vs. control group, ^##^*P* < 0.01 versus CFA group, Figure 4(c)).
  ③ Changes of the expressions of adaptive immune cytokines in the ST36 acupoint of the rats during MA treatment. IL-2, IL-12, IFN-*γ*, IL-4, IL-5, IL-10, IL-13, and IL-17 are some commonly encountered cytokines of adaptive immunity. There was no significant effect on their expressions in CFA + MA group compared with that in the CFA and control group at day 1, day 7, and day 15 (Figure 5).
  ④ Changes of the several growth factors expressions in the ST36 acupoint of the rats during MA treatment. GM-CSF, G-CSF, and M-CSF VEGF EPO are several growth factors included in the Rat Cytokine 24-Plex Assay kit. Compared with CFA group, the concentration of GM-CSF in ST36 acupoint in CFA + MA group decreased significantly at day 1 (^#^*P* < 0.05, Figure 6(a)).

In summary, the key cytokines of the ST36 acupoint at three different MA time-points screened by statistical method are summarized in Table 1.

### 3.3. MA Dynamically Regulated the Immune Cytokines Connection Network in ST36 Acupoint of the Rats with CFA-Induced Inflammation

**Figure 7:**
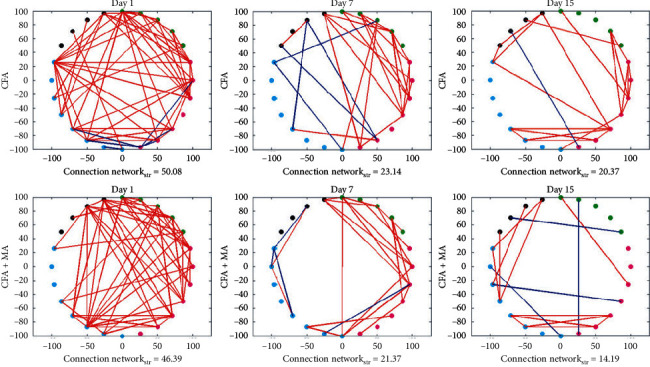
Cytokine association network of ST36 acupoint in groups on days 1, 7, and 15. Cytokine association network (correlation coefficients ∈ [−1, −0.8], [0.8, 1]) of the ST36 acupoint in CFA group and CFA + MA group. Green dots represent chemokines, pink dots represent innate immune cytokines, blue dots represent adaptive immune cytokines, and black dots represent growth factors. Red lines represent positive correlation between the cytokines, and blue lines represent negative correlation between the cytokines. The thicker line represents greater correlation coefficient, and the thinner line represents smaller correlation coefficient. (B) CCC network diagram.

Correlation coefficients between cytokines in the local ST36 acupoint were calculated by Pearson correlation coefficient formula, and the immune cytokines connection networks (see Figure 7) were constructed based on the cytokines filtered by correlation coefficients ∈ [−1, −0.8], [0.8, 1]. By calculating the Connection network_str_ of CFA group and CFA + MA group at three different time-points, it was found that overall correlation intensity of cytokines was gradually decreased with time after CFA injection, while MA could make the overall correlation intensity even lower. Then the screened cytokines were sorted by node degree, node correlation intensities, and node clustering coefficient (see supplemental material Tables S1–S3). The first three nodes obtained by analysis with 2 or 3 methods mentioned above were considered as the key cytokines screened based on complex network analysis (see Table 2).

### 3.4. MA Dynamically Regulated the Immune Cell-Cell Communication Network in ST36 Acupoint of the Rats with CFA-Induced Inflammation

The secretory cells and target cells of 24 cytokines were obtained manually from the immunologic book [20], containing innate immune cells (monocyte macrophages, neutrophils, dendritic cells, natural killer cells (NK cells), natural killer T cells (NKT cells), mast cells, eosinophils and basophils), adaptive immune cells (CD4^+^T cells, TH1 cells, TH2 cells, TH9 cells, TH17 cells, TH22 cells, Treg cells, cytotoxic lymphocyte cells (CTL cells), type 1 regulatory T cell (Tr1 cells), B cells, and plasma cells), and body cells (endothelial cells, epithelial cells, fibroblast cells, keratinocyte cells, stromal cells, hypothalamic cells, liver cells, smooth muscle cells, and adipocyte cells).

**Figure 8:**
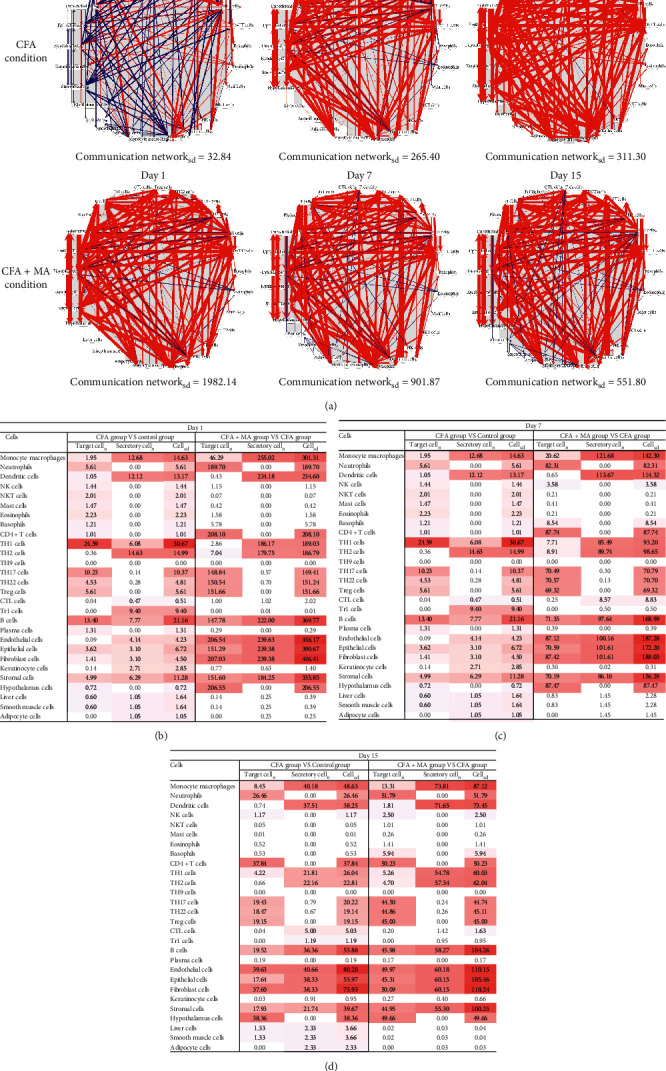
CCC network diagram of ST36 acupoint in groups on days 1, 7, and 15. (a) CCC network diagram. The CCC network diagram was mapped in model condition induced by CFA compared with the control group (CFA condition) and the condition of CFA rats treated with MA compared with CFA group (CFA + MA condition). Communication network_sd_ represents the whole immune network density between cells in indicated condition. (b–d) Heat maps of total density of involved cells on days 1, 7, 15, target cell_*n*_, secretory cell_*n*_, and cell_sd_ with high value (deep color) were considered as the key cells in the communication network of ST36 acupoint.

The directed weighted CCC network was performed to determine the communication intensity between the involved cells. A high communication network_sd_ value represents a strong communication intensity. The communication network_sd_ in ST36 acupoint in CFA group was elevated with the time-course (network_sd_ 32.84 on day 1, 265.40 on day 7, and 311.30 on day 15), indicating that CFA injection enhanced the immunity state of ST36 acupoint (Figure 8(a)). While compared with CFA group, the communication network_sd_ of ST36 acupoint in CFA + MA group was more enhanced at the three time-points of day 1, day 7, and day 15 (network_sd_ 1982.14 on day 1, 901.87 on day 7, and 551.80 day 15); however, the intensity decreased gradually with time, which indicated that MA intervention could enhance the immune function of ST36 acupoint but it decreased with time. The target cell_n_, secretory cell_n_, and cell_sd_ values of each cell were calculated and analyzed by heat map (Figures 8(b)–8(d)). The cells with highest value (dark color) were considered to be the key cells in the CCC network. Final results were shown in Table 3; among them, macrophages with the highest secretion intensity and fibroblasts cells with the highest total cell intensity at day 1, day 7, and day 15 are more worthy of attention.

### 3.5. MA Increased the Expression of Macrophages at ST36 Acupoint in the Rats with CFA-Induced Inflammation

**Figure 9:**
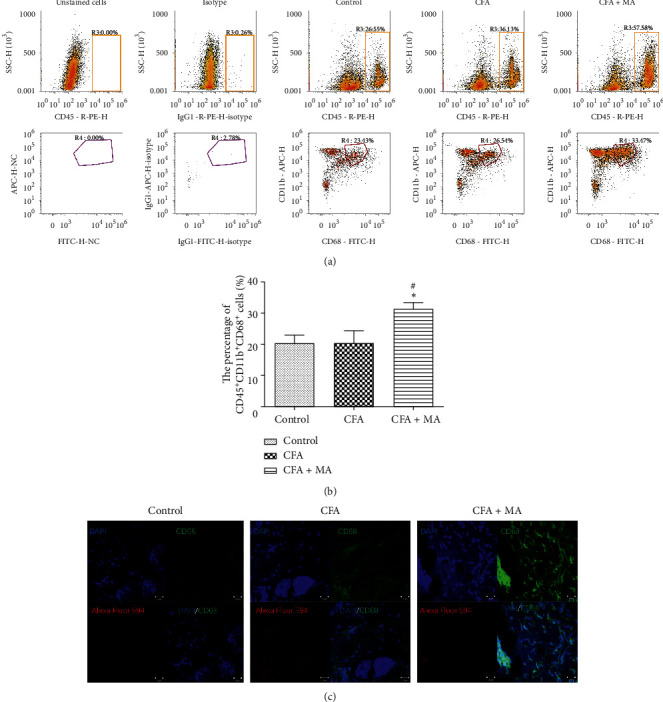
Changes in the macrophage level in ST36 acupoint after MA. Macrophages labeled with CD45^+^CD11b^+^ CD68^+^ levels in the skin layers of ST36 acupoint at day 7 were quantitatively detected through flow cytometry. Data are expressed as the mean ± SEM; ^*∗*^*P* < 0.05 vs. control group, ^#^*P* < 0.05 vs. CFA group (a, b). At the same time, macrophages labeled with CD68^+^ were positioned by immunofluorescence in skin layers (dermis) (c), scale bar: 20 *μ*m. In the control group and CFA group, the number of local macrophage expressions was low, while the expression was significantly increased after MA.

Based on the results of CCC network analysis, we detected the macrophage expression in local ST36 acupoint tissue at day 7 by flow cytometry and immunofluorescence technique, and then locational and quantitative analysis were carried out. The results showed that the number of macrophages labeled with CD45^+^CD11b^+^CD68^+^ was less in the control group and CFA group, but it increased significantly in CFA + MA group (^*∗*^*P* < 0.05 vs. control group, ^#^*P* < 0.05 vs. CFA group, Figures 9(a) and 9(b)). The results of immunofluorescence assay showed the increased macrophages were mainly distributed in the dermis of acupoint skin in CFA + MA group (Figure 9(c)).

## 4. Discussion

The present study confirmed that MA has an analgesic, detumescence, and anti-inflammatory effect on rats with inflammatory pain, which was consistent with our previous study [16] and other researches [21, 22]. MA regulated the network connection between cytokines at local ST36 acupoint and reduced the overall correlation intensity, while strengthening the correlation degree of the key cytokines; moreover, MA reinforced communication intensity between immune cells and enhanced the immune function. More importantly, cytokines IL-6, MCP-1, fibroblasts cell, and monocyte macrophage at ST36 acupoint screened by statistics, complex network, and CCC diagram methods at three MA time-points might be the key cytokines and key cells and play a very important role in the initiation process of MA effect. These findings provide a basis for revealing the initial mechanism of MA effect.

The statistical results of 24 cytokines detection showed that MA could significantly increase the concentration of the key cytokines MCP-1, CXCL1, MIP-3 *α*, IL-1 *β*, and IL-6 in ST36 acupoint of the rats with inflammatory pain, which was consistent with some of the results of our previous studies [12]; however, it has not shown significant effects on adaptive immune cytokines and several common growth factors. CXCL1, MCP-1, and MIP-3*α* belong to chemokines, which can attract macrophages, neutrophils, and other cells to migrate to inflammatory sites and that is important in inflammatory reaction [23]. IL-1 *β* and IL-6 belong to the cytokines of innate immunity, are produced by monocyte macrophages, fibroblasts, and other cells in the state of infection and inflammation, which have many biological functions, such as participating in the regulation of immunity and the metabolism of the body, and especially play an important role in promoting the occurrence and development of inflammation. These results implied that as a traumatic physical stimulation, MA may activate and regulate the immune system, especially the innate immune system but has little effect on adaptive immunity and growth factor. It might be because of the large dispersion of the data at day 7, the key cytokines screened at day 1 and day 15 in CFA + MA group had no statistical difference compared with CFA group at day 7 time-point, and it is necessary to expand sample size in order to confirm the experimental results in the future.

The results of cytokines connection network suggest that MA can regulate the network connection between cytokines at ST36 acupoint and reduce the overall correlation intensity while strengthening the correlation degree of the key cytokines; this reflects the regulatory effect of MA to restore homeostasis [24], and the key cytokines might affect other cytokines. Furthermore, the key cytokines were screened out by complex network analysis. Among them, TNF- *α*, as an important initiating factor in inflammatory response, can induce waterfall release of cytokines such as IL-1 and IL-6 and promote leukocyte aggregation and activation in inflammatory sites. The main function of IL-5 is to stimulate the proliferation, differentiation, and activation of eosinophils. M-CSF and G-CSF can stimulate the colony formation of granulocytes and macrophage and promote their function. However, the role of these key cytokines at different MA time-points still needs to be studied.

Our previous study [12] showed as a mechanical stimulus that MA could cause local tissue damage or microtrauma of the acupoints. So we speculated the microtrauma of the acupoints induced by MA can activate the immune regulation. The results of CCC network showed MA could enhance the immune function of ST36 acupoint, but it decreased with the passage of time. It indicated the local microtrauma could enhance the local immune function of acupoints in the early stage of MA, participating in the effect of MA. However, if this immune response is infinitely enhanced, it will lead to excessive immunity at acupoints and cause harm to the body. Therefore, in the late stage of MA, the role of enhancing local immune function at acupoints is gradually weakened, eventually exerting the effect of MA and protecting the local tissues at acupoints, which accords with the basic characteristics of MA [24]. Monocyte macrophages and fibroblasts cells, the key cells screened out at three MA time-points, are critical in the pathophysiological processes of tissue trauma, repair, and inflammation. Researchers have studied the relationship between local fibroblasts cells in acupoint and acupuncture analgesia and found that the activation of the ERK on skin fibroblasts cells in acupoint of mice with inflammatory pain is the key factor inducing the effect of acupuncture analgesia [5]. Another study confirms that the adenosine in local acupoint mediates the analgesic effect of acupuncture, and the adenosine mainly comes from ATP degradation in the cytoplasm of muscle cells and fibroblasts [25].This shows that fibroblasts at the acupoints have an important influence on the acupuncture effect. Therefore, in this experiment, to verify the other key cell screened out by CCC diagram methods, we detected the macrophage in the ST36 acupoint by flow cytometry and immunofluorescence and found that the relative amount of macrophages increased significantly after MA, especially the macrophage of the dermis of the skin. The key cytokines screened by statistics and cytokine association network were obviously secreted by macrophages, which indicated that macrophages at acupoints might the key cells involved in the initiation of the analgesic effect of MA on inflammatory pain, and these cells might cooperate with key cytokines to amplify the information of MA.

Summarizing all the results of this experiment, we believe that MA could significantly increase the concentration of the key cytokines MCP-1, CXCL1, MIP-3*α*, IL-1*β*, and IL-6 in ST36 acupoint of rats with inflammatory pain at three MA time-points, reduced the overall correlation intensity between cytokines, but at the same time enhanced the correlation of the key cytokines IL-5, IL-6, M-CSF, MCP-1, IL-1 *β*, G-CSF, and TNF-*α* with other cytokines. MA could also enhance the local immune function, especially increasing the expression of macrophages and promoting the activation of the key cells such as monocyte macrophages, fibroblasts cells, CD4+ T cells, and neutrophils. The above key cytokines and key cells might interact with or promote each other, further activate other molecular and cellular functions, activate the “acupoint network,” and amplify local information of MA, thus triggering the overall regulation of MA.

Many interesting and valuable results have been found in this study, but there were still some limitations. Firstly, the study did not set up a sham MA control group, so it is not certain that the current results are specific to MA. Secondly, only one animal model was used in this experiment, which limited the extensibility of the results. Thirdly, for the screened key cells, this experiment only detected macrophages at one MA time-point but did not verify other time-points and other key cells, which should be supplemented in future research. Fourthly, we only focused on the immune-related cytokines and cells in the local MA acupoints; other changes such as hormones and neuropeptides have not been paid attention and should be further studied. Fifthly, although the experimental animal samples of behavioral testing were more abundant, the sample size of 24 cytokines concentration detection, macrophage localization, and quantitative detection was still small, and it is necessary to expand the sample size to further affirm the experimental results. Finally, the following questions need to be further studied; that is, how do the changes of key cells and key cytokines in the acupoints cause the overall regulation of MA to achieve the effect of analgesic, detumescence, and anti-inflammatory? What is the specific mechanism? We will explore them in future research.

## 5. Conclusion

The present study confirmed the key cytokines and immune cells in local acupoint involved in MA treating inflammatory pain. This work improves our understanding of the scientific basis underlying acupoint initial priming mechanism of MA analgesia.

## Figures and Tables

**Table 1:** Key cytokines in ST36 acupoint based on statistical analysis.

	Day 1	Day 7	Day 15
CFA + MA group	MCP-1	—	MCP-1
	CXCL1	—	CXCL1
	IL-1*β*	—	MIP-3*α*
	IL-6	—	IL-1*β*
		—	IL-6

**Table 2:** Key cytokines in ST36 acupoint based on complex network analysis.

	Day 1	Day 7	Day 15
CFA group	IFN-*γ*	MIP-1*α*	IL-6
	MCP-1	TNF-*α*	IL-7
	IL-1*α*	CXCL1	IL-1*β*

CFA + MA group	IL-5	IL-6	IL-6
	M-CSF	MCP-1	IL-1*β*
	G-CSF	M-CSF	TNF-*α*

**Table 3:** Key cells in ST36 acupoint based on CCC network diagram analysis.

		Target cell	Secretory cell	Cell_sd_
CFA condition	Day 1	TH1 cells	TH2 cells	TH1 cells
	Day 7	TH1 cells	TH2 cells	TH1 cells
	Day 15	Endothelial cells	Endothelial cells	Endothelial cells

CFA + MA condition	Day 1	CD4^+^T cells	Monocyte macrophages	Fibroblast cells
	Day 7	CD4^+^T cells	Monocyte macrophages	Fibroblast cells
	Day 15	Neutrophils	Monocyte macrophages	Fibroblast cells

## Data Availability

The data used to support the findings of this study are available from the corresponding author upon request.

## Supplementary Materials

Supplementary Materials Table S1: cytokine in ST36 acupoint sorted by complex network analysis on day 1. Table S2: cytokine in ST36 acupoint sorted by complex network analysis on day 7. Table S3: cytokine in ST36 acupoint sorted by complex network analysis on day 15.
