# Supplementary material for: Applying Complex Network and Cell-Cell Communication Network Diagram Methods to Explore the Key Cytokines and Immune Cells in Local Acupoint Involved in Acupuncture Treating Inflammatory Pain
Source: Evid Based Complement Alternat Med. 2020 Jul 29;2020:2585960. doi: 10.1155/2020/2585960 (PMC7411476; doi:10.1155/2020/2585960)
Supplement: Supplementary Materials — Table S1: cytokine in ST36 acupoint sorted by complex network analysis on day 1. Table S2: cytokine in ST36 acupoint sorted by complex network analysis on day 7. Table S3: cytokine in ST36 acupoint sorted by complex network analysis on day 15. [file 2585960.f1.docx]

**Supplemental material**

**Table S1.** **Cytokine in ST36 acupoint sorted by complex network analysis on day1**

| D1 | Molecular  sorting | Node  degree | Molecular  sorting | Node strength  correlations | Molecular  sorting | Node clustering  coefficient |
| --- | --- | --- | --- | --- | --- | --- |
| CFA | IFN-γ | 12 | IFN-γ | 10.64 | TNF-α | 0.00 |
|  | MCP-1 | 10 | MCP-1 | 8.70 | IL-2 | 0.00 |
|  | IL-1β | 8 | IL-1α | 7.29 | IL-4 | 0.00 |
|  | IL-1α | 8 | IL-1β | 7.23 | IL-17 | 0.00 |
|  | IL-6 | 7 | IL-5 | 6.58 | GM-CSF | 0.00 |
|  | IL-7 | 7 | IL-18 | 6.53 | VEGF | 0.00 |
|  | IL-18 | 7 | IL-10 | 6.52 | G-CSF | 0.00 |
|  | IL-5 | 7 | IL-7 | 6.47 | IL-12 | 0.33 |
|  | IL-10 | 7 | IL-6 | 6.36 | M-CSF | 0.38 |
|  | M-CSF | 7 | M-CSF | 6.04 | IFN-γ | 0.45 |
|  | CXCL1 | 6 | CXCL1 | 5.50 | MCP-1 | 0.56 |
|  | MIP-3α | 5 | MIP-3α | 4.35 | IL-1β | 0.57 |
|  | CRP | 5 | CRP | 4.28 | IL-6 | 0.67 |
|  | IL-12 | 4 | IL-12 | 3.47 | MIP-3α | 0.70 |
|  | MIP-1α | 3 | MIP-1α | 2.72 | CXCL1 | 0.73 |
|  | RANTES | 2 | IL-13 | 1.69 | IL-1α | 0.75 |
|  | IL-2 | 2 | IL-2 | 1.66 | IL-7 | 0.90 |
|  | IL-13 | 2 | RANTES | 1.66 | IL-18 | 0.90 |
|  | IL-4 | 1 | IL-4 | 0.86 | IL-5 | 0.90 |
|  | VEGF | 1 | G-CSF | 0.82 | IL-10 | 0.90 |
|  | G-CSF | 1 | VEGF | 0.80 | MIP-1α | 1.00 |
|  | TNF-α | 0 | TNF-α | 0.00 | RANTES | 1.00 |
|  | IL-17 | 0 | IL-17 | 0.00 | CRP | 1.00 |
|  | GM-CSF | 0 | GM-CSF | 0.00 | IL-13 | 1.00 |
| CFA+MA | IL-5 | 11 | IL-5 | 9.78 | CRP | 0.00 |
|  | G-CSF | 9 | M-CSF | 8.07 | IL-2 | 0.00 |
|  | M-CSF | 9 | G-CSF | 7.95 | IL-13 | 0.00 |
|  | IL-1β | 8 | IL-1β | 6.89 | IL-17 | 0.00 |
|  | MIP-3α | 7 | IL-7 | 6.33 | GM-CSF | 0.00 |
|  | TNF-α | 7 | TNF-α | 6.23 | VEGF | 0.00 |
|  | IL-7 | 7 | MIP-3α | 6.02 | MIP-3α | 0.43 |
|  | MCP-1 | 6 | MIP-1α | 5.42 | IL-5 | 0.47 |
|  | MIP-1α | 6 | MCP-1 | 5.37 | CXCL1 | 0.50 |
|  | IL-18 | 5 | IL-10 | 4.68 | IL-1β | 0.50 |
|  | IL-10 | 5 | IL-18 | 4.66 | G-CSF | 0.50 |
|  | CXCL1 | 4 | IL-4 | 3.63 | M-CSF | 0.58 |
|  | RANTES | 4 | RANTES | 3.51 | MCP-1 | 0.60 |
|  | IL-4 | 4 | CXCL1 | 3.37 | IL-7 | 0.62 |
|  | IL-1α | 3 | IL-6 | 2.84 | RANTES | 0.67 |
|  | IL-6 | 3 | IL-12 | 2.74 | TNF-α | 0.67 |
|  | IL-12 | 3 | IL-1α | 2.65 | IL-4 | 0.67 |
|  | IFN-γ | 2 | IFN-γ | 1.82 | MIP-1α | 0.80 |
|  | VEGF | 1 | VEGF | 0.82 | IL-1α | 1.00 |
|  | CRP | 0 | CRP | 0.00 | IL-6 | 1.00 |
|  | IL-2 | 0 | IL-2 | 0.00 | IL-18 | 1.00 |
|  | IL-13 | 0 | IL-13 | 0.00 | IL-10 | 1.00 |
|  | IL-17 | 0 | IL-17 | 0.00 | IL-12 | 1.00 |
|  | GM-CSF | 0 | GM-CSF | 0.00 | IFN-γ | 1.00 |

**Table S2. Cytokine in ST36 acupoint sorted by complex network analysis on day7**

| D7 | Molecular  sorting | Node  degree | Molecular  sorting | Node strength  correlations | Molecular  sorting | Node clustering  coefficient |
| --- | --- | --- | --- | --- | --- | --- |
| CFA | MIP-1α | 6 | MIP-1α | 5.49 | RANTES | 0.00 |
|  | TNF-α | 6 | TNF-α | 5.36 | IL-7 | 0.00 |
|  | CXCL1 | 4 | CXCL1 | 3.81 | IL-4 | 0.00 |
|  | IL-1β | 4 | IL-1β | 3.79 | IL-5 | 0.00 |
|  | IL-6 | 4 | IL-6 | 3.70 | IL-12 | 0.00 |
|  | M-CSF | 4 | M-CSF | 3.49 | IL-13 | 0.00 |
|  | MIP-3α | 3 | G-CSF | 2.67 | IL-17 | 0.00 |
|  | IL-18 | 3 | IL-18 | 2.65 | VEGF | 0.00 |
|  | CRP | 3 | CRP | 2.59 | MIP-3α | 0.33 |
|  | G-CSF | 3 | MIP-3α | 2.58 | CRP | 0.33 |
|  | MCP-1 | 2 | IL-10 | 1.84 | TNF-α | 0.40 |
|  | IL-1α | 2 | IL-1α | 1.72 | MIP-1α | 0.47 |
|  | IL-2 | 2 | IFN-γ | 1.67 | M-CSF | 0.50 |
|  | IL-10 | 2 | GM-CSF | 1.66 | IL-6 | 0.67 |
|  | IFN-γ | 2 | MCP-1 | 1.63 | IL-18 | 0.67 |
|  | GM-CSF | 2 | IL-2 | 1.63 | G-CSF | 0.67 |
|  | RANTES | 0 | RANTES | 0.00 | CXCL1 | 0.83 |
|  | IL-7 | 0 | IL-7 | 0.00 | IL-1β | 0.83 |
|  | IL-4 | 0 | IL-4 | 0.00 | MCP-1 | 1.00 |
|  | IL-5 | 0 | IL-5 | 0.00 | IL-1α | 1.00 |
|  | IL-12 | 0 | IL-12 | 0.00 | IL-2 | 1.00 |
|  | IL-13 | 0 | IL-13 | 0.00 | IL-10 | 1.00 |
|  | IL-17 | 0 | IL-17 | 0.00 | IFN-γ | 1.00 |
|  | VEGF | 0 | VEGF | 0.00 | GM-CSF | 1.00 |
| CFA+MA | IL-6 | 6 | IL-6 | 5.11 | CXCL1 | 0.00 |
|  | MCP-1 | 5 | MCP-1 | 4.37 | RANTES | 0.00 |
|  | MIP-3α | 4 | M-CSF | 3.44 | IL-1β | 0.00 |
|  | IL-1α | 4 | IL-1α | 3.40 | IL-7 | 0.00 |
|  | IL-2 | 4 | MIP-3α | 3.37 | IL-18 | 0.00 |
|  | M-CSF | 4 | IL-2 | 3.35 | CRP | 0.00 |
|  | TNF-α | 3 | IL-17 | 2.60 | IL-4 | 0.00 |
|  | IL-17 | 3 | IFN-γ | 2.56 | IL-5 | 0.00 |
|  | IFN-γ | 3 | TNF-α | 2.48 | IL-12 | 0.00 |
|  | MIP-1α | 2 | MIP-1α | 1.78 | IL-13 | 0.00 |
|  | IL-5 | 2 | G-CSF | 1.76 | GM-CSF | 0.00 |
|  | IL-10 | 2 | IL-10 | 1.63 | VEGF | 0.00 |
|  | G-CSF | 2 | IL-5 | 1.63 | IL-6 | 0.20 |
|  | CXCL1 | 1 | CXCL1 | 0.97 | TNF-α | 0.33 |
|  | RANTES | 1 | IL-1β | 0.97 | IL-2 | 0.33 |
|  | IL-1β | 1 | CRP | 0.86 | MIP-3α | 0.50 |
|  | IL-7 | 1 | IL-4 | 0.83 | MCP-1 | 0.60 |
|  | CRP | 1 | IL-7 | 0.82 | IL-1α | 0.67 |
|  | IL-4 | 1 | RANTES | 0.81 | IL-17 | 0.67 |
|  | IL-18 | 0 | IL-18 | 0.00 | IFN-γ | 0.67 |
|  | IL-12 | 0 | IL-12 | 0.00 | M-CSF | 0.67 |
|  | IL-13 | 0 | IL-13 | 0.00 | MIP-1α | 1.00 |
|  | GM-CSF | 0 | GM-CSF | 0.00 | IL-10 | 1.00 |
|  | VEGF | 0 | VEGF | 0.00 | G-CSF | 1.00 |

**Table S3. Cytokine in ST36 acupoint sorted by complex network analysis on day15**

| D15 | Molecular  sorting | Node  degree | Molecular  sorting | Node strength  correlations | Molecular  sorting | Node clustering  coefficient |
| --- | --- | --- | --- | --- | --- | --- |
| CFA | IL-6 | 5 | IL-6 | 4.51 | MCP-1 | 0.00 |
|  | IL-7 | 5 | IL-7 | 4.37 | CXCL1 | 0.00 |
|  | IL-1β | 4 | IL-1β | 3.72 | MIP-3α | 0.00 |
|  | TNF-α | 4 | TNF-α | 3.35 | RANTES | 0.00 |
|  | MIP-1α | 3 | MIP-1α | 2.81 | CRP | 0.00 |
|  | IL-18 | 3 | IL-18 | 2.79 | IL-2 | 0.00 |
|  | L-5 | 3 | L-5 | 2.69 | IL-4 | 0.00 |
|  | IL-10 | 3 | IFN-γ | 2.65 | IL-12 | 0.00 |
|  | IFN-γ | 3 | M-CSF | 2.65 | IL-13 | 0.00 |
|  | M-CSF | 3 | IL-10 | 2.63 | IL-17 | 0.00 |
|  | IL-1α | 2 | IL-1α | 1.81 | VEGF | 0.00 |
|  | GM-CSF | 2 | GM-CSF | 1.69 | G-CSF | 0.00 |
|  | MCP-1 | 1 | CRP | 0.87 | IL-6 | 0.40 |
|  | RANTES | 1 | VEGF | 0.87 | IL-7 | 0.40 |
|  | CRP | 1 | RANTES | 0.83 | TNF-α | 0.50 |
|  | IL-2 | 1 | G-CSF | 0.83 | IL-1β | 0.67 |
|  | VEGF | 1 | MCP-1 | 0.83 | IFN-γ | 0.67 |
|  | G-CSF | 1 | IL-2 | 0.83 | M-CSF | 0.67 |
|  | CXCL1 | 0 | CXCL1 | 0.00 | MIP-1α | 1.00 |
|  | MIP-3α | 0 | MIP-3α | 0.00 | IL-1α | 1.00 |
|  | IL-4 | 0 | IL-4 | 0.00 | IL-18 | 1.00 |
|  | IL-12 | 0 | IL-12 | 0.00 | L-5 | 1.00 |
|  | IL-13 | 0 | IL-13 | 0.00 | IL-10 | 1.00 |
|  | IL-17 | 0 | IL-17 | 0.00 | GM-CSF | 1.00 |
| CFA+MA | IL-6 | 5 | IL-18 | 2.82 | MCP-1 | 0.00 |
|  | IL-7 | 5 | IL-10 | 2.73 | CXCL1 | 0.00 |
|  | IL-1β | 4 | L-5 | 2.73 | MIP-3α | 0.00 |
|  | TNF-α | 4 | IL-7 | 2.68 | MIP-1α | 0.00 |
|  | MIP-1α | 3 | IL-12 | 2.49 | RANTES | 0.00 |
|  | IL-18 | 3 | IFN-γ | 1.82 | IL-1β | 0.00 |
|  | L-5 | 3 | M-CSF | 1.82 | IL-1α | 0.00 |
|  | IL-10 | 3 | IL-13 | 1.69 | IL-6 | 0.00 |
|  | IFN-γ | 3 | MCP-1 | 0.95 | TNF-α | 0.00 |
|  | M-CSF | 3 | IL-6 | 0.95 | CRP | 0.00 |
|  | IL-1α | 2 | CXCL1 | 0.89 | IL-2 | 0.00 |
|  | GM-CSF | 2 | CRP | 0.89 | IL-4 | 0.00 |
|  | MCP-1 | 1 | GM-CSF | 0.86 | IL-13 | 0.00 |
|  | RANTES | 1 | IL-2 | 0.85 | IL-17 | 0.00 |
|  | CRP | 1 | IL-17 | 0.85 | GM-CSF | 0.00 |
|  | IL-2 | 1 | TNF-α | 0.84 | VEGF | 0.00 |
|  | VEGF | 1 | G-CSF | 0.84 | G-CSF | 0.00 |
|  | G-CSF | 1 | RANTES | 0.84 | IL-12 | 0.33 |
|  | CXCL1 | 0 | VEGF | 0.84 | IL-7 | 1.00 |
|  | MIP-3α | 0 | MIP-3α | 0.00 | IL-18 | 1.00 |
|  | IL-4 | 0 | MIP-1α | 0.00 | L-5 | 1.00 |
|  | IL-12 | 0 | IL-1β | 0.00 | IL-10 | 1.00 |
|  | IL-13 | 0 | IL-1α | 0.00 | IFN-γ | 1.00 |
|  | IL-17 | 0 | IL-4 | 0.00 | M-CSF | 1.00 |
